# Excessive Islet NO Generation in Type 2 Diabetic GK Rats Coincides with Abnormal Hormone Secretion and Is Counteracted by GLP-1

**DOI:** 10.1371/journal.pone.0002165

**Published:** 2008-05-14

**Authors:** Albert Salehi, Sandra Meidute Abaraviciene, Javier Jimenez-Feltstrom, Claes-Göran Östenson, Suad Efendic, Ingmar Lundquist

**Affiliations:** 1 Department of Clinical Science, Universitetssjukhuset Malmö Allmäna Sjukhus (UMAS), Division of Endocrine Pharmacology, Karolinska Institute, Stockholm, Sweden; 2 University of Lund, Department of Molecular Medicine, Karolinska Institute, Stockholm, Sweden; 3 Department of Experimental Medical Science, Karolinska Institute, Stockholm, Sweden; Mayo Clinic College of Medicine, United States of America

## Abstract

**Background:**

A distinctive feature of type 2 diabetes is inability of insulin-secreting β-cells to properly respond to elevated glucose eventually leading to β-cell failure. We have hypothesized that an abnormally increased NO production in the pancreatic islets might be an important factor in the pathogenesis of β-cell dysfunction.

**Principal Findings:**

We show now that islets of type 2 spontaneous diabetes in GK rats display excessive NO generation associated with abnormal iNOS expression in insulin and glucagon cells, increased ncNOS activity, impaired glucose-stimulated insulin release, glucagon hypersecretion, and impaired glucose-induced glucagon suppression. Pharmacological blockade of islet NO production by the NOS inhibitor N^G^-nitro-L-arginine methyl ester (L-NAME) greatly improved hormone secretion from GK islets suggesting islet NOS activity being an important target to inactivate for amelioration of islet cell function. The incretin hormone GLP-1, which is used in clinical practice suppressed iNOS and ncNOS expression and activity with almost full restoration of insulin release and partial restoration of glucagon release. GLP-1 suppression of iNOS expression was reversed by PKA inhibition but unaffected by the proteasome inhibitor MG132. Injection of glucose plus GLP-1 in the diabetic rats showed that GLP-1 amplified the insulin response but induced a transient increase and then a poor depression of glucagon.

**Conclusion:**

The results suggest that abnormally increased NO production within islet cells is a significant player in the pathogenesis of type 2 diabetes being counteracted by GLP-1 through PKA-dependent, nonproteasomal mechanisms.

## Introduction

Diabetes type 2 is now a global health problem, characterized of both insulin resistance and impaired insulin response to glucose, defects that are regarded multifactorial in origin and are considered a result of both environmental and undefined genetic factors. We have shown [1], [2], [3], [4], [5], [6], [7], [8] that islet nitric oxide (NO) derived from neuronal constitutive NO synthase (ncNOS) is a strong negative modulator of glucose-stimulated insulin release. This enzyme resides abundantly in β-cells [9]. Moreover, ncNOS is in part associated with insulin granules [10] making it a suitable regulator of the secretory process. We have also found, in healthy animals, that exposure of islets to high concentrations of glucose or lipids induces expression and strong activity of inducible NOS (iNOS) in their β-cells concomitant with reduced insulin response to glucose [2], [3], [7], [11]. These data suggested to us that glucose-induced “glucotoxicity” as well as lipid-induced “lipotoxicity” in the β-cell, at least in part, might be due to possible nonimmunogenic deleterious effects of iNOS-derived NO. Hence there is reason to believe that a sustained elevation of islet iNOS activity and the ensuing excessive production of NO might be of significant importance in the development of type 2 diabetes.

Nutrient ingestion stimulates secretion of gut hormones such as glucagon-like peptide-1 (GLP-1) and gastric inhibitory polypeptide (GIP), which serve to amplify glucose-stimulated insulin release [12], [13]. GLP-1 is also known to decrease glucagon release [12], [13]. Both effects are glucose-dependent and GLP-1 is considered therapeutically useful to reduce elevated blood glucose [12], [13]. Therefore, GLP-1 has received great attention as treatment for type 2 diabetes [12], [13], [14]. We found recently that GLP-1 restored the impairment of glucose-stimulated insulin release in islets taken from lipid-infused rats and that this effect was linked to increased cyclic AMP levels and suppression of islet activities of both ncNOS and iNOS [3]. Since surprisingly even short-time (∼60 min) exposure of islets to high glucose in healthy animals was associated with enhanced production of iNOS-derived NO [7], [11], the present investigation was undertaken to study islet NOS activities and the influence of GLP-1 on an animal model of spontaneous type 2 diabetes and “glucotoxicity”, the mildly diabetic Goto-Kakizaki (GK) rat, which is considered a good model of human type 2 nonobese diabetes [15], [16], [17], [18]. Because the glucagon-producing α-cells harbor the ncNOS enzyme [9], which apparently is an important regulator of glucagon release [1], [2], [3], [4], [6], [18], [19], [20], [21], and because raised plasma levels of glucagon is a common feature of and contribute to human type 2 diabetes [22] we also performed parallel studies on glucagon secretion.

## Results

### Confocal microscopy of iNOS expression in islets directly isolated from GK rats

**Figure 1:**
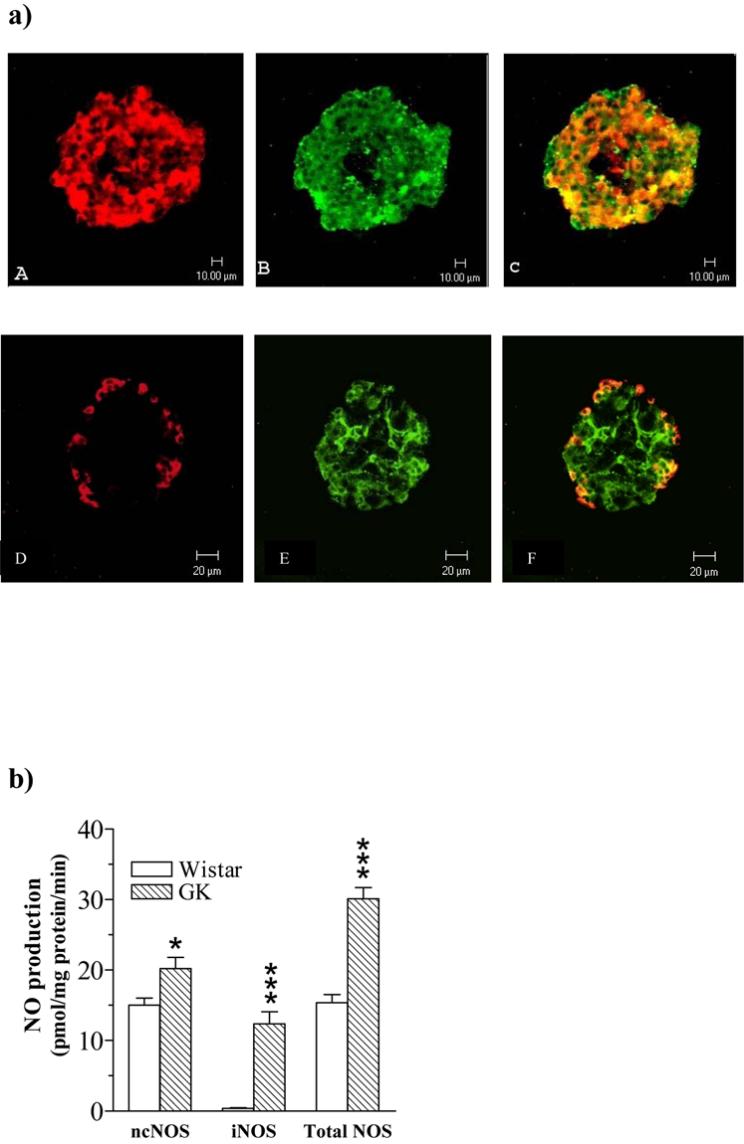
a Confocal microscopy of islets directly isolated ex vivo from the GK rat. The islets were double-immunolabelled for insulin or glucagon and iNOS and analysed by confocal microscopy. Insulin staining and iNOS staining appear, respectively, as *red* (A) and *green* (B) fluorescence. Co-localization of insulin/iNOS is seen as *orange-yellowish* fluorescence (C). Similarly glucagon staining and iNOS staining appear, respectively, as *red* (D) and *green* (E) fluorescence. Co-localization of glucagon/iNOS is seen as *orange-yellowish* fluorescence (F). Bars indicate lengths (10 µm). b Plates G–I and J–L show the absence of iNOS fluorescence in Wistar control islets (H, K). c *NOS activities in freshly isolated islets.* NO production from ncNOS, iNOS and total NOS in freshly isolated islets from Wistar control rats (open bars) and GK rats (hatched bars). Values are mean±s.e.m for n = 4–6 animals. *P<0.05; *** P<0.001

To investigate whether isolated islets express iNOS protein we performed a confocal microscopic study in GK islets. Islets were simultaneously immunolabeled for insulin or glucagon to investigate β- and α-cell specific expression of iNOS. As shown in **Fig. 1a**, immunoreactivity for iNOS was detected in insulin immunoreactive β-cells (A–C) and in glucagon immunoreactive α-cells (D–F) (orange-yellowish fluorescence in the overlay pictures). No iNOS immunoreactivity was found in Wistar control islets (**Fig. 1b**). Insulin immunoreactive β-cells (G–I) and glucagons immunoreactive α-cells (J–L) are shown.

### NOS activities in islets directly isolated from GK and Wistar rats

**Fig. 1c** shows that islets isolated from GK rats displayed increased ncNOS activity. Moreover, while iNOS activity in Wistar islets was negligible, GK islets displayed high iNOS activity. Hence total NO generation was impressively increased in GK compared with Wistar islets (**Fig. 1c**).

### Effect of the NOS inhibitor L-NAME on insulin and glucagon secretion from isolated Wistar and GK islets at low and high glucose and in vivo effect of L-arginine

To directly study the basic involvement and regulatory role of islet NO production for insulin and glucagon release we incubated isolated islets from GK and Wistar rats at low and high glucose in the absence and presence of the NOS inhibitor L-NAME (5 mmol/l). **Table 1a** shows that glucose-stimulated insulin release from both GK and Wistar islets is markedly amplified in the presence of L-NAME. This amplification is more pronounced in GK islets (90%) compared with Wistar islets (38%). No effect of L-NAME on insulin release was seen at low glucose (**Table 1a**). Moreover, NOS inhibition by L-NAME greatly suppressed the glucagon hypersecretion from GK islets incubated at low glucose (**Table 1b**). Such an inhibition of glucagon release from GK islets in the presence of L-NAME could also be seen at high glucose. Further, L-NAME did decrease glucagon secretion also from Wistar islets at low glucose, but had no significant effect at high glucose (**Table 1b**). Finally, to further test a regulatory role of ncNOS for a stimulated acute release of insulin and glucagon *in vivo* we injected L-arginine, which is known to stimulate both insulin and glucagon secretion, in the absence and presence of L-NAME pretreatment. **Table 1c and d** shows that NOS inhibition amplified the insulin but decreased the glucagon response following L-arginine injection in both GK and Wistar rats..

**Table 1:** Effect of the NOS inhibitor L-NAME on insulin and glucagon release *in vitro* and *in vivo.*

		Wistar	GK
**Insulin release (ng/islet per h)**			
**a)**	1G	0.269±0.029	0.258±0.032
	1G+L-NAME	0.305±0.052	0.303±0.033
	16.7G	4.01±0.32	1.89±0.26
	16.7G+L-NAME	5.61±0.35 **	3.68±0.38 ***
**Glucagon release (pg/islet per h)**			
**b)**	1G	29.46±1.1	41.04±1.62
	1G+L-NAME	20.04±1.3 **	29.38±1.7 **
	16.7G	18.33±1.9	36.64±2.25
	16.7G+L-NAME	15.67±2.1	26.35±2.53 **
**Plasma insulin response (pmol/l)**			
**c)**	Saline+L-arg.	948±111	238±54
	L-NAME+L-arg.	1349±82 **	410±52 *
**Plasma glucagon response (ng/l)**			
**d)**	Saline+L-arg.	551±45	404±71
	L-NAME+L-arg.	331±67 *	214±36 *

Effect of pharmacological blockade of islet NO generation by the NOS inhibitor L-NAME on islet hormone secretion from Wistar and GK rats. **a)** Insulin release and **b)** glucagon release from isolated islets at low and high glucose in the absence or presence of L-NAME (5 mmol/l). n = 8 in each group. Asterisks denote significant effects of L-NAME at 1G or 16.7G.

* p<0.05;

** p<0.01;

*** p<0.001.

**c)** Peak insulin response and **d)** Peak glucagon response in plasma at 2 min after an *i.v.* injection of L-arginine (L-arg.) (3.6 mmol/kg) following pretreatment with saline or L-NAME (1.2 mmol/kg). There were 4–7 animals in each group. Asterisks denote significant effects of L-NAME pretreatment.

* p<0.05;

** p<0.01

### Influence of GLP-1 on basal insulin and glucagon release in relation to islet ncNOS and iNOS expression and activities in incubated islets from GK and Wistar rats

**Figure 2:**
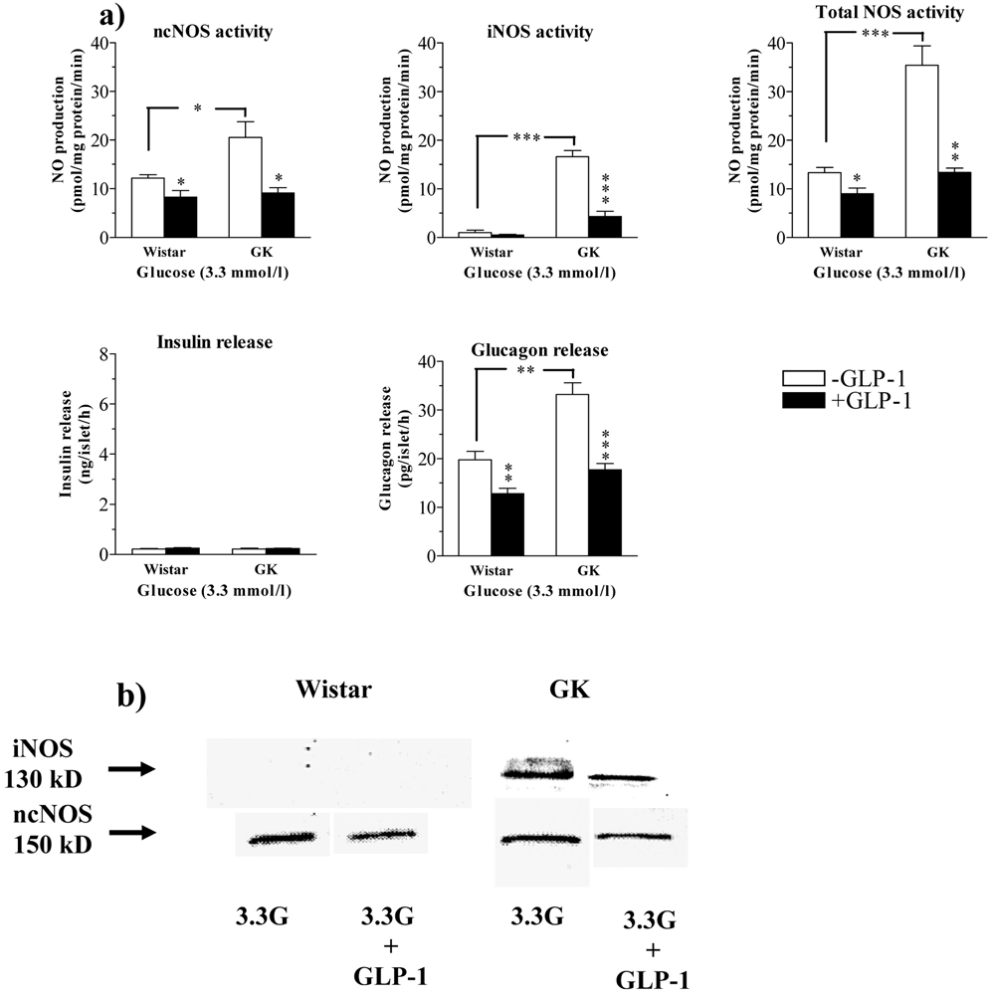
(a) NOS activities and hormone secretion in islets incubated at low glucose. Islet NO production from ncNOS, iNOS and total NOS as well as insulin and glucagon release from islets of Wistar or GK rats incubated at 3.3 mmol/l glucose in the absence (open bars) and presence (dark bars) of 100 nmol/l GLP-1. Values are mean±s.e.m for 5–9 batches of islets at each point. *P<0.05; ** P<0.01; *** P<0.001. (b) Representative examples of Western blots of iNOS and ncNOS protein in the absence and presence of GLP-1 are shown.

Since not only pharmacological blockade [5], [6], [8], [10], [11] but also cyclic AMP stimulating agents are known to suppress islet NO production in healthy animals[3], [7], we tested possible beneficial effects of GLP-1 on islet NOS activities in the GK rat. **Fig. 2 a, b** and **Table 2a** show the effect of GLP-1 on islet NOS activities and protein expression (Western blot) as well as insulin and glucagon secretion in islets from GK and Wistar rats incubated at low glucose (3.3 mmol/l). We used a concentration of 100 nmol/l of GLP-1, having maximal stimulating effect on glucose-induced insulin release in isolated rat islets [3]. Total NO generation was markedly increased in GK islets (**Fig. 2a**). This was mainly due to iNOS activity. No significant iNOS expression and activity was detectable in Wistar islets (**Fig. 2a, b**). ncNOS activity was modestly upregulated in GK islets (**Fig. 2a**). GLP-1 induced pronounced suppression of iNOS expression and activity in GK islets and suppressed ncNOS activity in both types of islets (**Fig. 2a, b** and **Table 2a**). Basal insulin secretion in GK and Wistar islets was similar at low glucose and GLP-1 had no effect (**Fig. 2a**). Glucagon secretion was impressively increased in GK islets *vs* Wistar islets (33.2±2.4 pg/islet per h *vs* 19.8±1.7 pg/islet per h; p<0.01) (**Fig. 2a**). GLP-1 suppressed glucagon secretion to 17.2±1.3 pg/islet per h in GK islets and to 12.8±1.1 pg/islet per h in Wistar controls. Notably, GK islets still hypersecreted glucagon after GLP-1 treatment (**Fig. 2b**). The densitometric analysis showed that GLP-1 induced a pronounced suppression of both ncNOS and iNOS expression in GK islets. A marked suppression of ncNOS expression was found also in Wistar islets, while no iNOS expression could be detected (**Table 2a**).

**Table 2:** Densitomeric analysis of iNOS and ncNOS protein expression.

	Wistar		GK	
**a)**	**3.3G**	**3.3G+GLP-1**	**3.3G**	**3.3G+GLP-1**
iNOS	0.2±0.09	0.13±0.05	18.7±3.3	8.5±1.1 ***
ncNOS	15.5±1.5	10.1±1.2 *	22.9±3.1	7.6±2.3 ***
**b)**	**16.7G**	**16.7G+GLP-1**	**16.7G**	**16.7G+GLP-1**
iNOS	16.3±2.05	6.1±0.91 ***	24.5±2.8	10.4±2.9 ***
ncNOS	25.2±2.4	15.05±1.7 **	40.5±4.1	11.3±2.01 ***

Densitometric analysis of Western blots for iNOS and ncNOS expression after incubation of Wistar and GK islets in the absence and presence of GLP-1 (100 nmol/l) at **a)** low glucose, 3.3 mmol/l (3.3G) and **b)** high glucose, 16.7 mmol/l (16.7 G). Asterisks denote significant effect of GLP-1 for n = 4 in each group.

* p<0.05;

*** p<0.001.

### Confocal microscopic study of the effects of GLP-1, the PKA inhibitor H-89 and the proteasome inhibitor MG 132 on iNOS expression in GK islets incubated at low glucose

**Figure 3:**
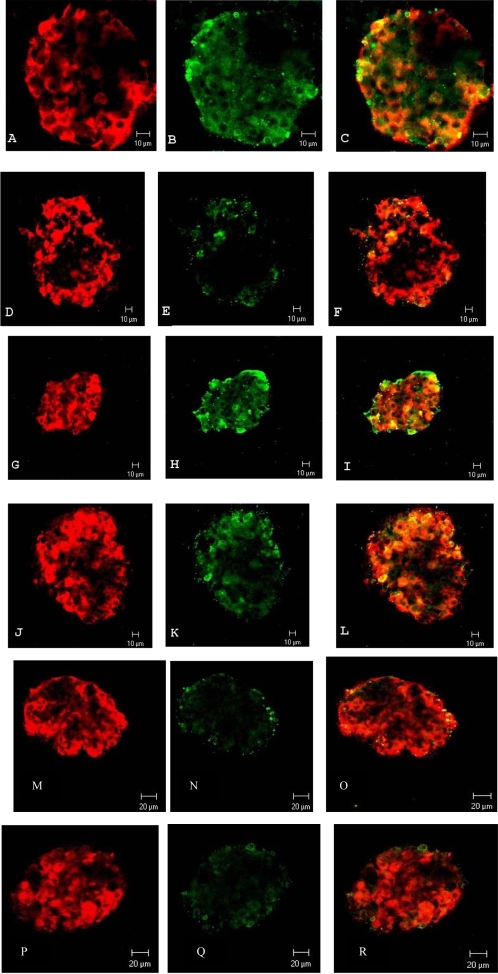
Confocal microscopy of incubated islets from the GK rat. Isolated islets were incubated for 90 min in the presence of; A, B and C) 3.3 mmol/l glucose; D, E and F) 3.3 mmol/l glucose+100 nmol/l GLP-1; G, H and I) 3.3 mmol/l glucose+100 nmol/l GLP-1+2 µmol/l H-89; J, K and L) 3.3 mmol/l glucose+2 µmol/l H-89; M, N and O) 3.3 mmol/l glucose+100 nmol/l GLP-1+10 µmol/l MG 132; P, Q and R) 3.3 mmol/l glucose+10 µmol/l MG 132. After incubation the islets were double immunolabeled for insulin and iNOS and analysed by confocal microscopy. Insulin and iNOS stainings appear, respectively, as *red* (A, D, G, J, M and P) and *green* (B, E, H, K, N and Q) fluorescence. Co-localisation of insulin/iNOS is seen as a *orange-yellowish* fluorescence (C, F, I, L, O and R). Plates S-U shows Wistar control islets at 3.3 mmol/l glucose. No iNOS expression could be detected (T). Bars indicate lengths (10 µm).

The suppressive effect by GLP-1 on iNOS protein expression in islets from healthy rats incubated at high glucose is PKA-mediated [7]. Because iNOS expression was present already in directly isolated GK islets next experiments were conducted at low glucose to avoid interference with *in vitro* glucose-induced iNOS stimulation [7]. Thus immunolabeling of iNOS expression at 3.3 mmol/l glucose was performed and effects of GLP-1 and the PKA inhibitor H-89 were recorded. GK islets were simultaneously immunolabeled for insulin to identify β-cell specific iNOS expression. As shown in **Fig. 3** A–C, iNOS immunoreactivity was detected in insulin-immunoreactive β-cells. GLP-1 (100 nmol/l) greatly suppressed the expression of iNOS (**Fig. 3** D–F) and the PKA inhibitor H-89 (2 µmol/l) reversed this suppression (**Fig. 3** G–I). **Fig. 3** J–L shows that H-89 by its own has no apparent effect on iNOS expression. Finally we studied the possible involvement of the proteasome system, since the proteasome has been suggested to modulate iNOS expression in other cell types [23], [24]. **Fig. 3** M–O shows that the suppressive effect of GLP-1 on iNOS expression (**Fig. 3** D–F) was not reversed by the proteasome inhibitor MG 132 (10 µmol/l), and **Fig 3** P–R shows, surprisingly, that the prominent iNOS expression in the β-cells (**Fig. 3** A–C) was abolished also after incubation with MG 132 itself. **Fig. 3** S–U shows that no iNOS expression was found in Wistar control islets.

### Influence of GLP-1 on insulin and glucagon release in relation to islet ncNOS and iNOS expression and activities in islets from GK and Wistar rats incubated at high glucose

**Figure 4:**
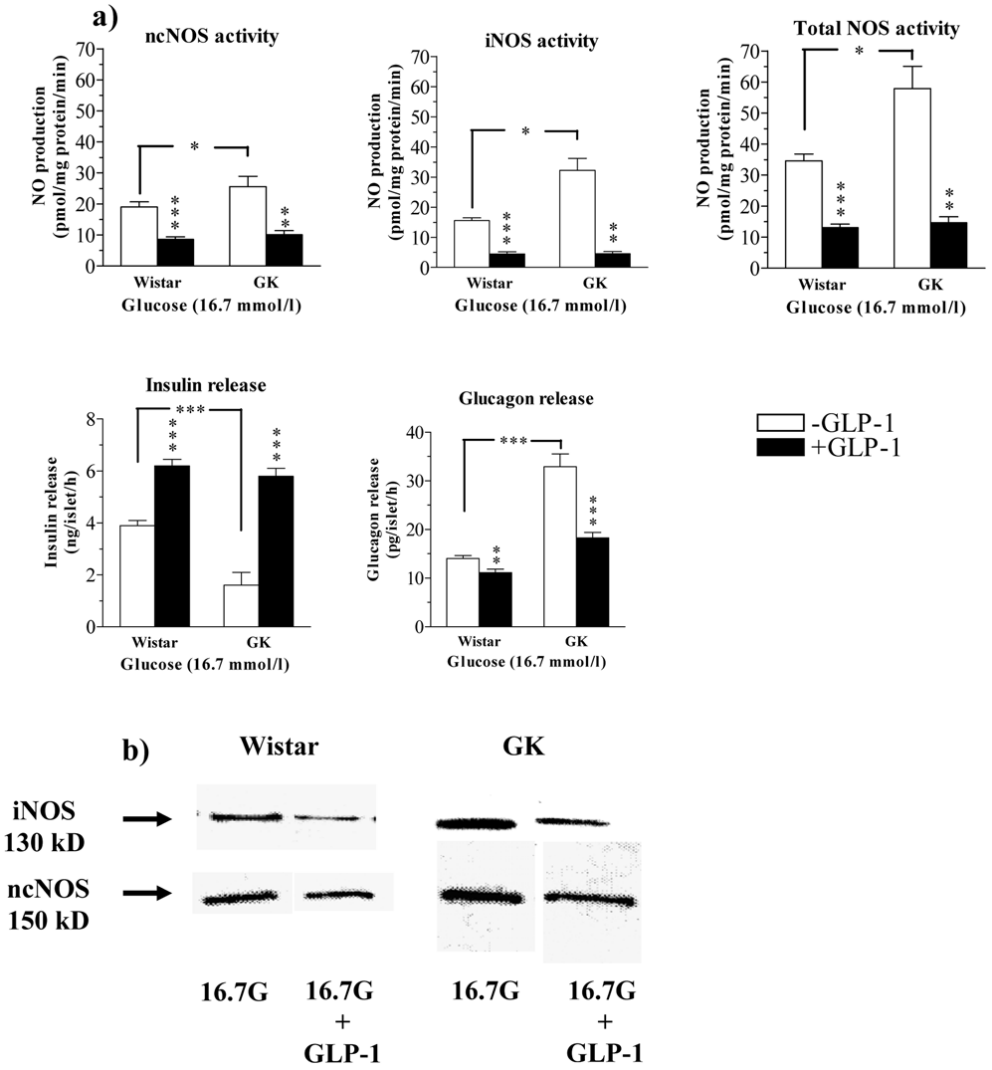
(a) NOS activities and hormone secretion in islets incubated at high glucose. Islet NO-production from ncNOS, iNOS and total NOS as well as insulin and glucagon release from islets of Wistar or GK rats incubated at 16.7 mmol/l glucose in the absence (open bars) and presence (dark bars) of 100 nmol/l GLP-1. Values are mean±s.e.m for 6–10 batches of islets at each point. *P<0.05; ** P<0.01; *** P<0.001. (b) Representative examples of Western blots of iNOS and ncNOS protein in the absence and presence of GLP-1 are shown.

**Fig. 4a, b** and **Table 2b** shows the effect of GLP-1 on NOS activities and protein expression in islets incubated at high glucose (16.7 mmol/l). Again, NOS activities were increased in GK compared with Wistar islets. This was mainly due to increased iNOS activity (**Fig. 4a**). However, upregulation of NOS activities was not so pronounced in GK islets at high glucose as at low glucose possibly because NOS activities were already upregulated *in vivo* and in comparison high glucose increased NOS activities also in Wistar islets (**Fig. 4a, b**). NO production and Western blots show that GLP-1 reduced both expression and activities of iNOS and ncNOS (**Fig. 4a, b**, **Table 2b**). This suppression was associated with a prominent increase in glucose-stimulated insulin release and thus the impairment of glucose-induced insulin response in GK islets was efficiently counteracted (**Fig. 4a**). Moreover, while high glucose suppressed glucagon secretion in Wistar islets, negligible suppression by glucose was found in glucagon hypersecreting GK islets (compare **Fig. 2a** and **4a**). GLP-1 reduced glucagon secretion in both types of islets (**Fig. 2a**). However, compared to Wistar, GK islets still displayed elevated glucagon secretion even after combination of high glucose and GLP-1 (**Fig. 4a**). The densitometric analysis showed that GLP-1 induced a marked suppression of both ncNOS and iNOS expression in GK as well as Wistar islets (**Table 2b**).

### Basal plasma levels of insulin, glucagon and glucose in GK and Wistar rats and effects of glucose and GLP-1 injections

**Figure 5:**
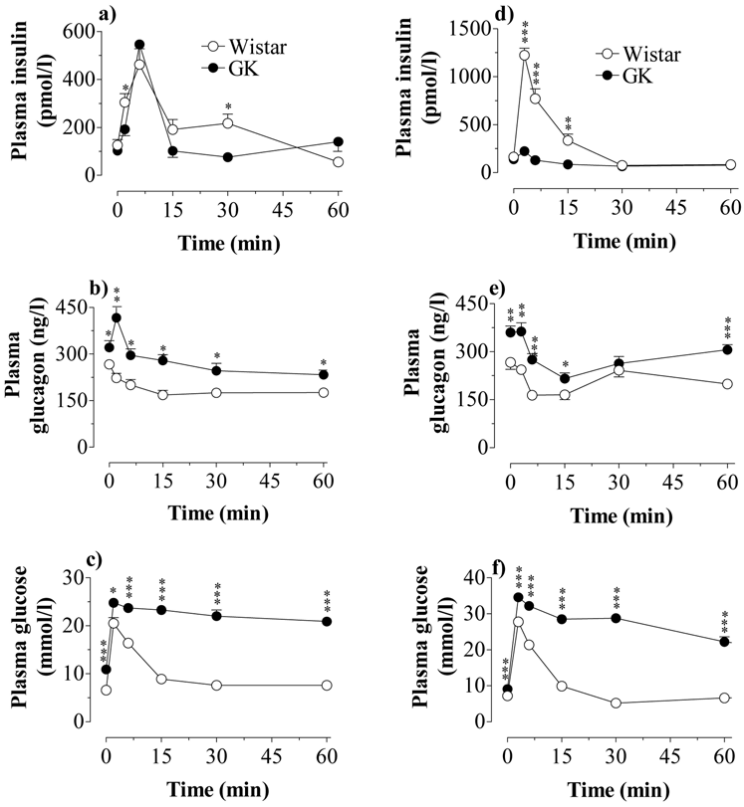
In vivo action of GLP-1 and glucose. Effect of an *iv* injection of GLP-1 (10 nmol/kg)+glucose (4.4 mmol/kg)(a–c) or glucose alone (11.1 mmol/kg)(d–f) on the plasma concentrations of insulin, glucagon and glucose in Wistar and GK rats. Values are mean±s.e.m for 8 animals in each group. *P<0.05; ** P<0.01; *** P<0.001.

Since GK islets apparently hypersecreted glucagon we measured circulating levels of glucagon, insulin and glucose *in vivo*. In addition we measured islet content of glucagon and insulin. Plasma levels of glucagon and glucose were elevated in GK rats, while insulin levels were not appreciably different. However, calculating the basal insulinogenic index, *i.e.* dividing circulating insulin concentrations with circulating glucose concentrations, revealed that the insulin response to basal glucose was impaired in GK *vs* Wistar rats (data not shown). The plasma levels were as follows, Wistar *vs* GK: Insulin (pmol/l) 100±8 *vs* 81±6.4 (NS); Glucagon (ng/l) 254.5±10.2 *vs* 311.5±12.9 (p<0.001); Glucose (mmol/l) 7.3±0.2 *vs* 11.0±0.2 (p<0.001). There were 28 animals in each group. Islet content of insulin and glucagon at 6 weeks of age was similar in GK and Wistar rats. Insulin content Wistar *vs* GK was 5.5±0.6 and 5.6±0.9 nmol/mg protein and glucagon content 1.02±0.09 and 0.89±0.09 µg/mg protein (n = 8 in each group). Because the action of GLP-1 is known to be highly glucose-dependent [12], [13] we examined *in vivo* insulin and glucagon responses to GLP-1 mixed with glucose. **Fig. 5a–b** shows the effects of an *iv* injection of this mixture and a control experiment with a high dose of glucose alone (**Fig. 5d–e**). The insulin response to the combination of GLP-1 and glucose was modestly lower in GK compared with Wistar. However, there was an abnormal transient increase in glucagon response followed by a slow suppression, which did not reach the low levels of glucagon recorded in Wistar. After injection of glucose alone the insulin response in GK rats was abrogated, and the glucagon response showed an initial normal suppression but a marked rebound (**Fig. 5d–e**). An impaired glucose tolerance curve after glucose alone (**Fig. 5f**) was still impaired in the GK rat even after addition of GLP-1 (**Fig. 5c**).

## Discussion

Previous studies have suggested that high amounts of NO derived from iNOS activity are cytotoxic and implicated in the autoimmune-mediated dysfunction and destruction of islet β-cells during development of type 1 diabetes [25]. This iNOS-stimulated NO production is not only mediated by invading macrophages but also by the β-cells themselves [25]. On the other hand, the role of islet ncNOS, which produces small amounts of NO in a signaling way, is still unclear [1], [2], [3], [4], [5], [6], [7], [8], [10], [11], [18], [19], [20], [26], [27], [28], [29], [30]. We have found that inhibition of ncNOS activity is accompanied by increased insulin release induced by glucose, L-arginine and to a lesser extent by cholinergic stimulation, while secretion induced by agents directly stimulating the cyclic AMP system is not inhibited and might even be slightly increased by NO [1], [2], [3], [4], [5], [6], [7], [8], [11], [19], [20], [29]. Moreover, islet NOS activities are efficiently counteracted by the cyclic AMP/PKA system [2], [3], [7], [29].

Based on these observations we proposed that ncNOS-derived NO might serve as a physiological negative feedback inhibitor of acute glucose-stimulated insulin release [1], [3], [5], [7], [8], [11]. Furthermore, we have shown in healthy mice that hyperglycemia lasting for only ∼60 min results in islet iNOS expression and activity [11], a finding that raised the question whether the islet NO system, and especially iNOS, might be implicated also in the development of nonimmunogenic type 2 diabetes.

Our present results showed a rich occurrence of iNOS protein in both β-cells and α-cells of islets isolated from the diabetic GK rat. The abundance of iNOS expression in the α-cells of the GK rat was unexpected, since injection of large doses of iNOS-stimulating endotoxin did induce iNOS only in single α-cells while almost all β-cells were affected [9], [29]. This observation thus raised the question whether long-term hyperglycemia might contribute to nonimmunogenic diabetes by inducing iNOS in the α-cells. Because NO not only inhibits insulin release but also stimulates glucagon release [1], [2], [3], [6], [19], [20], [21], [29] this observation thus underlined a possible pathogenic role of the islet NO system in the development of type 2 diabetes. In accordance, GK islets displayed increased ncNOS activity and impressive iNOS activity compared with Wistar control islets. These findings might, at least in part, explain the defective insulin response to glucose and the glucagon hypersecretion in these rats and also a possible deleterious effect of iNOS-derived NO on the β-cells over time. Further, the present data showing an abnormally increased iNOS-derived NO production also after *in vitro* incubation of GK islets at low glucose suggest that moving the islets from an *in vivo* hyperglycemic milieu to a medium of a low “hypoglycemic” glucose concentration (3.3 mmol/l) for a period of ∼2 h did not reduce or abolish iNOS expression and activity.

The NOS inhibitor L-NAME has previously been shown to serve as a potent inhibitor of islet ncNOS activity both *in vitro* and *in vivo* in the rat as well as in the mouse [5], [6], [8], [10], [11], [19], [27], [31]. Such a pharmacological inhibition of β-cell ncNOS activity was now found to restore the impaired insulin response to glucose and also to restore, at least in part, the increase glucagons secretion in GK islets thus suggesting that the increased NO generation in these islets is indeed an important factor for β-cell dysfunction and α-cell hyper-responsiveness in this model of animal type 2 diabetes. These results were further strengthened by the *in vivo* L-arginine data showing that L-NAME amplified the insulin but decreased the glucagon response. This is in accordance with a recent study in a new rat model of type 2 diabetes induced by streptozotocin- nicotinamide [32]. These authors found that an impaired ncNOS expression was associated with insulin release hyper-responsiveness to L-arginine.

From previous studies [3], [6], [7], [29] we hypothesized that a protective mechanism against the NO-mediated negative effects on β-cell function in GK rats might be exerted through the cyclic AMP system. We therefore selected the potent cyclic AMP-generating incretin, GLP-1 as a suitable agent to test this hypothesis. In our present short-term experiments GLP-1 counteracted the impaired insulin response to glucose and induced a marked decrease in islet NOS activities. Since we have shown that increased islet endogenous NO production and addition of exogenous NO gas or an intracellular NO donor to incubated islets are accompanied by reduction of glucose-stimulated insulin response [1], [2], [3], [5], [8], [11], we are now inclined to ascribe, at least in part, the beneficial effect of GLP-1 on the impairment of acute glucose-induced insulin release in the GK rat to its suppressive action on the islet NO system. Apart from its effect on the cyclic GMP system, NO is known to affect multiple targets within the cell, mainly acting through S-nitrosylation [33], [34], [35], [36]. It is unclear whether cyclic GMP, the glutathione system and/or different regulatory proteins in the stimulus-secretion coupling are primarily affected [8], [11], [34], [35], [36], [37] and regulation of NOS activities has been observed at all levels from gene transcription to covalent modification and allosteric regulation of the enzyme itself [33]. Interestingly, recent results from brain tissue have demonstrated that a number of metabolic, structural, and signaling proteins might be afflicted by NO through S-nitrosylation processes [36]. Notably the present study was performed in very young GK rats, and thus a more or less continuous activity of iNOS-derived NO might have further deleterious effects on the β-cell in the long run and earlier studies showed that β-cell numbers were diminished in 6-months old GK rats [15]. Such a notion is further underlined by previous data showing marked diabetes in mice with iNOS overexpression in their β-cells [38]. It should be recalled that the β-cell mass in the Stockholm GK colony is not affected in young rats [39].

The possible implication of proteasomal mechanisms in the loss of iNOS protein during short-time islet incubation with GLP-1 was explored by using the proteasome inhibitor MG 132. Unexpectedly, MG 132 did not reverse the GLP-1-induced suppression of iNOS expression but instead induced loss of iNOS protein and hence proteasomal inhibition did not prevent but stimulated degradation of iNOS, suggesting other control mechanisms regulating the cellular balance of this protein. A similar paradoxical effect of proteasomal inhibition was reported concerning (pro)insulin, the degradation of which was increased by the proteasome inhibitor lactacystin [40]. Similarly, β-cell iNOS induced by lipid infusion to rats was abolished by treatment of isolated islets from such rats with MG 132 [41]. A paradoxical effect of proteasomal inhibition by MG 132 was also reported in vascular smooth muscle [23]. These observations raised the question whether certain regulatory mechanisms modulating the expression and degradation of iNOS might be circumvented by the lack of appropriate signaling through the mere presence of a proteasome inhibitor or by other less well-understood mechanisms possibly including factors affiliated with ER stress [42], [43]. In fact, very recent studies have shown a most complex series of events induced by various proteasomal inhibitors in different tissues[44], [45]. Thus proteasome inhibition indeed seems to induce ER stress followed by activation of autophagic processes in *e.g.* β-cells [44], embryonic fibroblasts and different cancer cell lines [45], while HEK 293 cells and macrophage cell line RAW 264.7 were dependent on the proteasome degradation pathway [24]. The present observation that proteasome inhibition in GK β-cells stimulates the degradation of iNOS protein within 90 min raises further questions concerning the complex interaction between the proteasomal and autophagic systems in the β-cell, which hopefully will be answered in future studies.

Our *in vivo* experiments showed that a combination of GLP-1 and glucose induced a fairly good insulin response in GK rats, while the response to glucose alone was abrogated. These results agreed with our *in vitro* data, showing a restraining action of GLP-1 on islet NOS activities, and a concomitant amplification of glucose-stimulated insulin release. These effects of GLP-1 are most likely attributed to its ability to increase the islet cyclic AMP/PKA system as previously suggested from our data in healthy animals [2], [3], [7]. Although also PKA-independent effects of cyclic AMP on distal steps in the stimulus-secretion coupling are described [46] our data favor PKA being of major importance for the regulation of islet NOS activities.

The basal plasma concentrations of glucagon in young GK rats were increased. Moreover, an abnormal secretion of glucagon in GK rats was detected after injecting GLP-1 plus glucose. An unexpected transient hypersecretory glucagon response was followed by a slight suppression, which was less pronounced compared with the marked decrease of glucagon observed in controls. Hence GLP-1 only in part restored the abnormal hypersecretion of glucagon in GK rats. Glucose alone, however, induced a normal initial transient fall of glucagon but this eas followed by a marked rebound at 60 min. An abnormal hypersecretion of glucagon from GK islets was also observed *in vitro*. Notably glucose had a negligible suppressive effect on glucagon secretion in isolated GK islets, while GLP-1 decreased glucagon release concomitant with decreased NO generation in both types of islets, although less efficient so in GK islets. These data thus suggest that GLP-1 has a good amplifying effect on glucose-stimulated insulin release but might be less efficient in strengthening glucose-induced glucagon suppression *in vivo* in this model of type 2 diabetes. NO is an important stimulator of glucagon secretion in healthy animals [1], [2], [3], [6], [19], [20], [21], [29], [47] and ncNOS resides in both β-cells and α-cells [9], while no expression of iNOS is observed. As mentioned above, in GK rats there is an abundance of peripherally located islet cells, which were iNOS positive and corresponded to glucagon cells showing, that iNOS indeed is highly expressed also in the α-cells of the diabetic rats. Increased levels of circulating glucagon are a common feature in human type 2 diabetes [22]. Hence the present data strongly suggest that an abnormally increased NO production in α-cells might contribute to the abnormal glucagon hypersecretion in diabetes. The mechanisms of a less efficient effect by GLP-1 to suppress glucagon secretion in diabetes remain unclear but further underline the importance of restoring glucagon hypersecretion in this disease.

In conclusion, we believe that the importance of deleterious effects of excessive islet NO production in nonimmunogenic type 2 diabetes has been seriously overlooked and might explain some if not all of the impairments of islet hormone secretion and β-cell survival. The present data show that abnormally increased expression and activities of islet NOS isoenzymes coincide with increased glucagon secretion and decreased glucose-stimulated insulin release and thus that an excessive NO production is an important contributing factor for the diabetic condition. GLP-1 can, only in part, counteract these abnormalities through activating the cyclic AMP/PKA system. Our data also suggest that high concentrations of circulating glucagon is important for the diabetic condition even in the presence of GLP-1, although this incretin hormone contributes to an increased insulin release through suppressing the NOS activities in the β-cell. These novel data on an NO-generated glucotoxic action in GK islets might be applicable, at least in part, to human type 2 diabetes and hopefully pave the way for new therapeutic interventions to reduce islet NO production.

## Materials and Methods

### Animals

Young age-matched male GK rats of the Stockholm colony bred at the Karolinska Institute and Wistar control rats (commercially available from B&K, Sollentuna, Sweden) 5–7 weeks old were used in all experiments. They were fed a standard pellet diet (B&K) and tap water *ad libitum*. Notably it is known that certain colonies of Wistar rats might have a defective insulin response to glucose. Ancillary experiments in our laboratory, however, have shown that the actual B&K Wistar rats display an insulin response comparable to that of age-matched Sprague-Dawley rats. The experiments were approved by the Ethical Committee for Animal Research at the University of Lund, Sweden.

### Chemicals

Bovine serum albumin was from ICN Biochemicals, High Wycombe, UK. Glucagon-Like Peptide-1 (7–36) amide (GLP-1) was from Peninsula Laboratories, Belmont, CA, USA. H-89 and MG 132 were from Calbiochem, La Jolla, CA, USA. The NOS inhibitor N^G^-nitro-L-arginine methyl ester hydrochloride (L-NAME) and all other drugs and chemicals were from Sigma Chemicals, St Louis, MO, USA or Merck AG, Darmstadt, Germany. Polyclonal rabbit anti-iNOS and HRP-conjugated goat anti-rabbit IgG was from StressGen Biotechnologies Corp, Victoria, BC, Canada. Cy2-conjugated anti-rabbit IgG and Cy5-conjugated anti-guinea pig IgG were from Jackson Immunoresearch Laboratories Inc, West Grove, PA, USA. The radioimmunoassay kits for insulin and glucagon determinations were obtained from Diagnostika (Falkenberg, Sweden) and Eurodiagnostica (Malmö, Sweden), respectively.

### Isolation of pancreatic islets

Preparation of isolated pancreatic islets was performed by retrograde injection of a collagenase solution *via* the bile-pancreatic duct as previously described [1]. Islets were then collected under a stereomicroscope at room temperature.

### In vitro experiments

The freshly isolated islets were preincubated for 30 min at 37°C in Krebs Ringer bicarbonate (KRB) buffer, pH 7.4, supplemented with 10 mmol/l HEPES, 0.1% bovine serum albumin, and 3.3 mmol/l glucose. The composition of the KRB buffer was (in mmol/l); NaCl 120, KCl 4.7, CaCl_2_ 2.54, KH_2_PO_4_ 1.2, Mg SO_4_ 1.2 and NaHCO_3_ 25. Each incubation vial contained 250 islets in 1.5 ml buffer solution (60 islets in 1.5 ml for confocal experiments) and was gassed with 95% O_2_-5% CO_2_ to obtain constant pH and oxygenation. After preincubation the buffer was changed to a medium containing the test agents, and the islets were incubated for 90 minutes. All incubations were performed at 37°C in an incubation box (30 cycles/min). Immediately after incubation, aliquots of the medium were removed for assay of insulin and glucagon [48], [49], [50] and the islets were prepared for measurement of NOS activities as described below.

### Immunofluorescence and confocal microscopy

Islets freshly isolated or collected after incubation in KRB buffer as described above were fixed with 4% formaldehyde, permeabilized with 5% Triton X-100, and unspecific sites blocked with 5% Normal Donkey Serum (Jackson Immunoresearch Laboratories Inc, West Grove, PA, USA). iNOS was detected with a rabbit-raised polyclonal anti-iNOS antibody (StressGen Biotechnologies Corp, Victoria, BC, Canada) (1∶100) in combination with Cy2-conjugated anti-rabbit IgG (Jackson Immunoresearch Laboratories Inc, West Grove, PA, USA). (1∶150). For staining of insulin and glucagon, islets were incubated with a guinea pig-raised anti-insulin antibody (1∶1000) and anti-glucagon antibody (1∶200) (Eurodiagnostica, Malmö, Sweden) followed by an incubation with a Cy5-conjugated either anti-guinea pig IgG antibody (insulin) or Cy5-conjugated anti-rabbit IgG antibody (glucagon) (Jackson Immunoresearch Laboratories Inc, West Grove, PA, USA) (1∶150). The fluorescence was visualized with a Zeiss LSM510 confocal microscope by sequentially scanning at (excitation/emission) 488/505-530 nm (Cy2) and 633/>650 nm (Cy5).

### Measurement of NOS activities

Isolated islets (batches of 250 islets in 1.5 ml of buffer solution) were either prepared for direct assay of NOS activities or preincubated for 30 min and then incubated for 90 min in KRB buffer as described above. Thereafter an aliquot of the medium was taken for the assay of insulin and glucagon. Islets were thoroughly washed and collected in ice-cold buffer (200 µl) containing HEPES (20.0 mmol/l), EDTA (0.50 mmol/l) and D, L-dithiothreitol (DTT) (1.0 mmol/l), pH 7.2, and stored at −20°C for subsequent NOS analysis [1], [11]. In brief, after sonication on ice, the buffer solution containing the islet homogenate was supplemented to contain also CaCl_2_ (0.45 mmol/l), calmodulin (25 U/ml), NADPH (2.0 mmol/l) and L-arginine (0.2 mmol/l) in a total volume of 450 µl for determination of total NOS. For the assay of iNOS another portion of the homogenate was incubated in the absence of both calmodulin and CaCl_2_ [11]. The homogenates were then incubated at 37°C under constant air bubbling (1.0 ml/min) for 180 minutes. Aliquots of the incubated medium (200 µl) were mixed with an equal volume of *o*-phtaldialdehyde reagent solution in a glass vial and then passed through an 1 ml Amprep CBA cation-exchange column for high-performance liquid chromatography (HPLC) analysis. The amount of L-citrulline formed (NO and L-citrulline are produced in equimolar concentrations) was then measured in a Hitachi F1000 fluorescence spectrophotometer (Merck, Darmstadt, Germany) as previously described [1], [11]. The resulting activity for iNOS was subtracted from total NOS activity to give the ncNOS activity [11]. Protein was determined with the Bradford method [51].

### Western blot analysis

Islets incubated as stated above for the assay of islet NOS activities were analyzed for immunoblotting. After incubation the islets were washed in Hanks' buffer and then suspended in 150 µl of 10 mmol/l Tris lysis buffer, pH 7.4, containing 0.5% Triton X-100, 0.5 mmol/l EDTA and 0.2 mmol/l PEFA block, frozen and sonicated on ice on the day of analysis [11]. The protein content of the supernatant was determined according to Bradford [51]. Homogenate samples representing 10 µg of total protein were run on 7.5% SDS-polyacrylamide gel (Bio-Rad, Hercules, CA, USA). After electrophoresis, proteins were transferred to nitrocellulose membranes (Bio-Rad). The membranes were blocked in LS-buffer (10 mmol/l Tris, pH 7.4, 100 mmol/l NaCl, 0.1% Tween-20) containing 5% non-fat dry milk powder for 40 min at 37°C. Incubation was performed with rabbit anti-mouse ncNOS (N-7155) and iNOS (N-7782) (1∶2000; Sigma, St Louis, MO, USA). After three washings in LS-buffer the membranes were finally incubated with a horseradish peroxidase-conjugated goat anti-rabbit antibody (1∶50000). Immunoreactivity was detected using an enhanced chemiluminescence reaction (Pierce, Rockford, IL, USA). The intensities of the bands were quantified by densitometry (Bio-Rad GS-710 Densitometer).

### In vivo studies

The rats were injected *i.v* with a mixture of GLP-1 (10 nmol/kg) and glucose (4.4 mmol/kg), or glucose alone (11.1 mmol/kg) and blood sampling was performed as described previously [17]. GLP-1 was dissolved in 0.9% NaCl-0.1% gelatine. In the L-arginine experiments L-NAME (1.2 mmol/kg) or saline (controls) was injected *i.v.* 10 seconds before the *i.v.* injection of L-arginine (3.6 mmol/kg). Both L-NAME and L-arginine were dissolved in 0.9% NaCl. The volume load was 5 µl/g rat.

### Determination of insulin, glucagon and glucose

Insulin and glucagon were determined with radioimmunoassays [48], [49], [50]. Glucose was determined with glucose oxidase.

### Statistics

Results are expressed as mean±s.e.m. Probability levels of random differences were determined by the unpaired Student's t-test or where applicable by analysis of variance followed by Tukey-Kramer's multiple comparison test.
