# Excessive Islet NO Generation in Type 2 Diabetic GK Rats Coincides with Abnormal Hormone Secretion and Is Counteracted by GLP-1

**DOI:** 10.1371/annotation/a72da6a5-a71a-4cc9-ae2b-2b95c0ec89b0

**Published:** 2008-06-03

**Authors:** Albert Salehi, Sandra Meidute Abaraviciene, Javier Jimenez-Feltstrom, Claes-Göran Östenson, Suad Efendic, Ingmar Lundquist

There was an error in all three author affiliations. The correct affiliations should appear as shown here:

1 Department of Clinical Science, Division of Endocrine Pharmacology, Malmö University Hospital, University of Lund, Lund, Sweden, 2 Department of Molecular Medicine, Karolinska Institute, Stockholm, Sweden, 3 Department of Experimental Medical Science, University of Lund, Lund, Sweden

