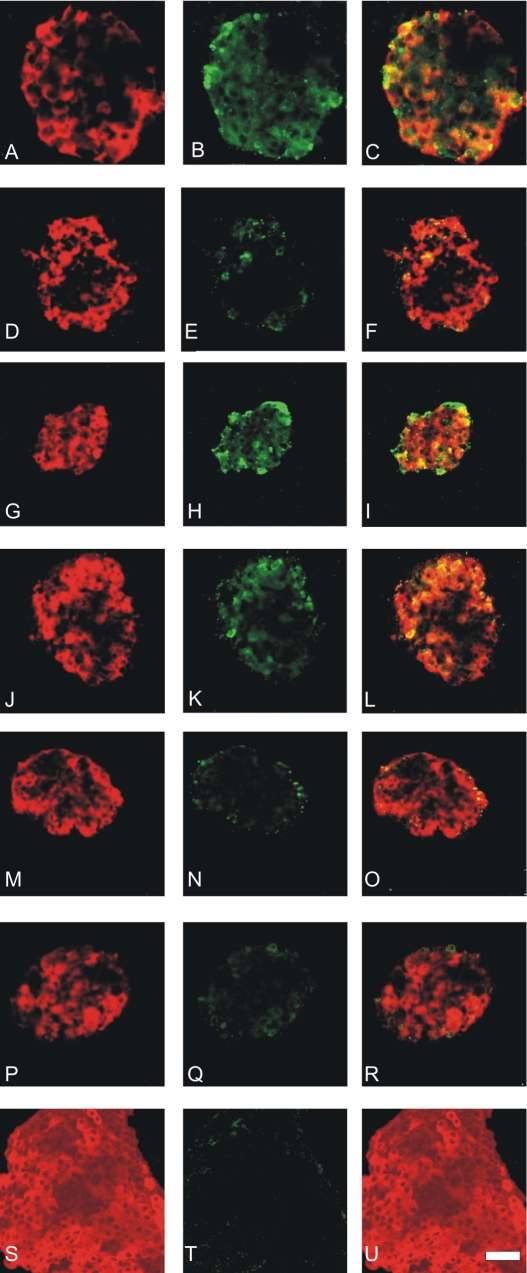# Correction: Excessive Islet NO Generation in Type 2 Diabetic GK Rats Coincides with Abnormal Hormone Secretion and Is Counteracted by GLP-1

**DOI:** 10.1371/annotation/5efa5d3b-2733-4629-9592-f41e0814ca0b

**Published:** 2008-06-03

**Authors:** Albert Salehi, Sandra Meidute Abaraviciene, Javier Jimenez-Feltstrom, Claes-Göran Östenson, Suad Efendic, Ingmar Lundquist

There was an in error in Figure 3. Panels (S-U) were not included. The corrected figure is available here:

**Figure:**