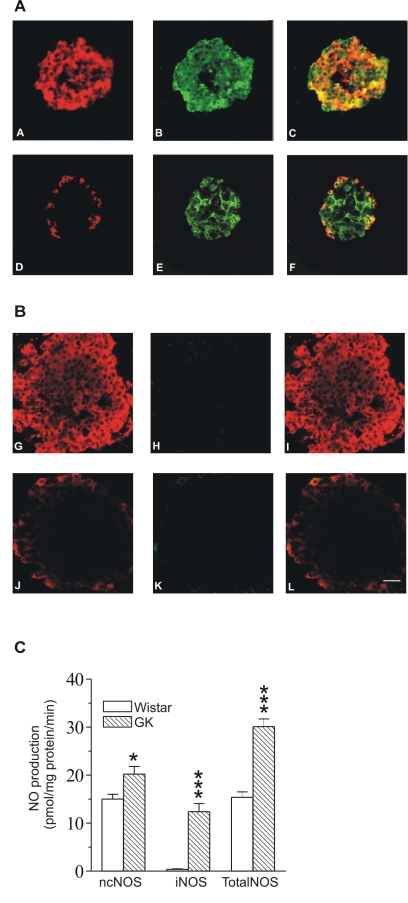# Correction: Excessive Islet NO Generation in Type 2 Diabetic GK Rats Coincides with Abnormal Hormone Secretion and Is Counteracted by GLP-1

**DOI:** 10.1371/annotation/bea154a4-93b3-4f1a-9948-acbaae12d256

**Published:** 2008-06-03

**Authors:** Albert Salehi, Sandra Meidute Abaraviciene, Javier Jimenez-Feltstrom, Claes-Göran Östenson, Suad Efendic, Ingmar Lundquist

There was an error in Figure 1. Panel (B) was not included and panel (C) was incorrectly labeled as (B). The corrected figure is available here:

**Figure:**